# Exploring lymphocyte subpopulations in children with behcet disease, a new diagnostic tool?

**DOI:** 10.1186/1546-0096-12-S1-P126

**Published:** 2014-09-17

**Authors:** Stefano Ghirardelli, Antonella Meini, Marco Cattalini, Daniele Moratto, Manuela Baronio, Alessandro Plebani, Vassilios Lougaris

**Affiliations:** 1 Pediatric Clinic, Department of Clinical and Experimental Sciences, University of Brescia, Brescia, Italy; 2 Laboratory for Molecular Medicine "A. Nocivelli", Spedali Civili di Brescia, Brescia, Italy

## Introduction

Behçet Disease (BD) is a chronic systemic vasculitis, with its typical onset during young adulthood. Pediatric onset of the disease may occur in a limited number of cases, and frequently the diagnostic criteria applied for adult patients may not be fully satisfied. Therefore in the last years new diagnostic criteria for pediatric-onset BD have been developed (PED-BDIC). The pathogenesis of BD is still not clear, although various immunological hypotheses have been proposed. In this regard, evaluation of peripheral lymphocyte subsets as indicators of the underlying immunological disturbance has been performed in a limited number of adult BD patients, but data in pediatric BD patients are lacking.

## Objectives

**T**he aim of this study was to investigate the peripheral lymphocyte subsets in pediatric BD, in order to evaluate if there exists an underlying immunological disturbance.

## Methods

30 children with BD followed at our Centre were included in this study. Patients were divided into 2 groups depending on the age at enrolment: <12 years of age (Group 1) and >12 years of age (Group 2); no differences in the disease duration in the two groups were noted. Absolute values of lymphocytes subpopulations from peripheral blood samples were analyzed using a flow cytometer FACSCalibur (Becton Dickinson, CA). Fluorescence data were acquired using the “Cell Quest Pro” software (Becton Dickinson, CA) and processed by “FlowJo” software (Tree Star, OR). Data were compared to age matched healthy donors (HD)

## Results

The distribution of peripheral lymphocyte subsets showed several differences between BD patients and HD. In particular in Group 1, CD4+ Naïve Central Memory and Effector Memory subpopulations were decreased when compared to HDs. CD8+ Total and Effector Memory T cells resulted also decreased when compared to HDs. Regarding B cells, an increase in Switched Memory B cells was noted, when compared to HDs. In Group 2, CD4+ Naïve and Central memory T cells were decreased when compared to HDs. CD8+ Total and Naïve T cells resulted reduced as well. B cells in Group 2 did not show any significant alterations. All the differences in lymphocyte subsets described reached statistical significance.

## Conclusion

We herein provide the first evidence of peripheral lymphocyte subsets disturbances in pediatric BD. These lymphocyte disturbances resulted more pronounced in younger BD patients (<12 years of age). Of interest, the majority of the data here described appear exclusive of the pediatric form of BD, since similar findings have not been reported in adult BD patients. Our findings may become useful during the diagnostic approach, especially in younger patients. Further studies in order to validate these results are ongoing.

## Disclosure of interest

None declared.

